# A Newcastle Programme

**Published:** 1921-07-30

**Authors:** 


### The Hospital

The Workers' Newspaper of Administrative Medicine and Institutional Life. Administration. National Insurance and Health.

No. 1834, Vol. LXX. SATURDAY, JULY 30, 1921. PRICE TWOPENCE

### A NEWCASTLE PROGRAMME.

The outstanding feature of the Newcastle meeting of the British Medical Association was decidedly the outspoken and courageous address of the new President, Dr. Drummond, who was inducted to office on the termination of Sir Clifford Albutt's occupation of the chair. Newspaper editors far and wide have fastened eagerly upon the one item most likely to tickle a public palate somewhat jaded by the heat of this amazing summer, the proposal that doctors should make *post-mortem* examinations at discretion, and not at the whim of sentimental and prejudiced relatives. This proposal was but one of a series of suggestions for improving the equipment of the medical profession in its campaign against disease and death; and it should be considered strictly upon its merits, and in due perspective as one small item amongst a number of others, some of them far more important. Yet certain newspapers have done more than concentrate the opinion of their public on this one topic alone: they have even gone out of their way to misrepresent Dr. Drummond as advocating compulsory *post-mortem* examination in every case, whereas he merely proposed that it should be compulsory only when the doctor in charge thinks it advisable or desirable.

The general thesis of the address—that is, the improvement of the education of the medical profession and of the medical practitioner, especially in the direction of detecting the first beginnings of divergence from health—is not novel: it has many times been urged in these columns, and as lately as July 9 our correspondent "Ierne" was dealing with it in our columns. Dr. Drummond has done good service to the public by re-emphasising it with all the *prestige* attaching to his position; and if his remarks about autopsies serve to focus public opinion on the larger question also, his object will have been well attained. Unfortunately the comments of the press do not seem to show an appreciation of the great difficulties which attach to the progress of research into the commencement of disease: it is most undesirable that the idea should get about that progress in this direction will be swift or easy, for disillusionment is quite certain to follow such dreams. That a beginning has been made by Sir James Mackenzie at St. Andrews, and that the medical profession is realising the task that lies ahead—these are facts of great promise for the future, but the performance may well absorb the energies of several generations yet, and a facile optimism is very distinctly out of place on the part of both the public and the doctors.

In respect of the specific question which has so intrigued the public press, that of autopsies, it is as well to clear the air of vague fallacies at once. It is absolutely sure that diagnosis, and, indeed, medical science as a whole, is enormously stimulated in hospital practice by the performance of autopsies there: it is recognised by everybody who has been in a medical school that both research and the training of medical students are immensely benefited by the proceedings in the *post-mortem* chamber. Be it remembered, however, that these autopsies are performed in the best possible circumstances by highly trained pathologists; and that in private practice conditions are far otherwise, and the possible benefits correspondingly reduced. At the same time there are cases, many cases, where a private practitioner wishes for an autopsy and could profit by it for the benefit of his future patients, but is deterred either by the opposition of the relatives or by his fear of incurring odium if he puts forward the suggestion. It would be, unquestionably, a public gain if the popular prejudice against autopsy, which is wholly irrational, could be overcome in the interest of medical science, which is, of course, the interest of every person alive. It is doubtful whether public opinion is ripe for so drastic a step as compulsion in the matter: one can imagine the philippics of all the political "outs" against the "ins" if such a proposal were put forward in Parliament by the Ministry of Health as a Government measure. Interesting and valuable, therefore, as the discussion of this point has been, it is of less importance at the moment than some other sections of Dr. Drummond's address which have been less commented upon. Of his far-sighted statesmanship and his devotion of great talents to the public welfare there are, in any case, no two opinions to be expressed, or have been, so far as we know.

